# The Interplay between Systolic Blood Pressure, Sauna Bathing, and Cardiovascular Mortality in Middle-Aged and Older Finnish Men: A Cohort Study

**DOI:** 10.1007/s12603-023-1895-1

**Published:** 2023-04-20

**Authors:** Jari A. Laukkanen, S.Y. Jae, J. Kauhanen, S.K. Kunutsor

**Affiliations:** 1 Institute of Public Health and Clinical Nutrition, University of Eastern Finland, Kuopio, Finland; 2 Institute of Clinical Medicine, Department of Medicine, University of Eastern Finland, P.O. Box 1627, FIN-70211, Kuopio, Finland; 3 Wellbeing Services County of Central Finland, Department of Medicine, Jyväskylä, Finland District, Jyväskylä, Finland; 4 Graduate School of Urban Public Health, University of Seoul, Seoul, Republic of Korea; 5 Department of Sport Science, University of Seoul, Seoul, South Korea; 6 Department of Urban Big Data Convergence, University of Seoul, Seoul, Republic of Korea; 7 National Institute for Health Research Bristol Biomedical Research Centre, University Hospitals Bristol NHS Foundation Trust and University of Bristol, Bristol, UK; 8 Musculoskeletal Research Unit, Translational Health Sciences, Bristol Medical School, University of Bristol, Southmead Hospital, Learning & Research Building (Level 1), BS10 5NB, Bristol, UK; 9 Diabetes Research Centre, University of Leicester, Leicester General Hospital, Gwendolen Road, LE5 4WP, Leicester, UK

**Keywords:** Cardiovascular disease, hypertension, mortality, sauna bathing, systolic blood pressure

## Abstract

**Objectives:**

Elevated systolic blood pressure (SBP) is associated with an increased risk of cardiovascular disease (CVD) mortality, whereas frequent sauna bathing reduces the risk. Whether frequent sauna bathing mitigates CVD mortality among adults with elevated SBP has not been previously investigated.

**Design and Setting:**

We examined the interactions between SBP and frequency of sauna bathing (FSB) with the risk of CVD mortality in a cohort of Caucasian men.

**Participants:**

The Kuopio Ischaemic Heart Disease Study cohort comprising of 2,575 men aged 42–61 years at baseline was employed for this prospective study analysis.

**Measurements:**

Resting blood pressure was measured using a standardized protocol and sauna bathing habits were assessed by a self-administered questionnaire. Systolic blood pressure was categorized as normal and high (<140 and ≥140 mmHg, respectively) and FSB as low and high (defined as ≤ 2 and 3–7 sessions/week, respectively).

**Results:**

A total of 744 CVD deaths were recorded during a median follow-up of 27.8 yr. Comparing high vs normal SBP, the multivariable-adjusted HR (95% CI) for CVD mortality was 1.44 (1.23–1.68). Comparing low vs high FSB, the multivariable-adjusted HR (95% CI) for CVD mortality was 1.24 (1.03–1.51). The associations persisted following mutual adjustment for each exposure. Compared with men with normal SBP-high FSB, high SBP-low FSB was associated with an increased risk of CVD mortality 1.81 (1.39–2.36), with attenuated but persisting evidence of an association for men with high SBP and high FSB 1.52 (1.06–2.16). When SBP was categorized as normal and high (<130 and ≥130 mmHg, respectively), there was no evidence of an association for men with high SBP and high FSB 1.11 (0.77–1.61).

**Conclusion:**

There might be an interaction between SBP, sauna bathing and CVD mortality risk in middle-aged and older Caucasian males. Frequent sauna baths may offset the increased risk of CVD mortality in men with high-normal SBP but not elevated SBP.

## Introduction

**T**hough established modifiable risk factors such as a blood pressure, history of diabetes, blood lipids, and smoking status explain a large proportion of the risk of cardiovascular disease (CVD) (1), it remains the leading cause of death globally (2). A wealth of epidemiological studies have reported associations between these individual risk factors and cardiovascular outcomes including mortality as well as global estimates of the impact of multiple modifiable risk factors on these outcomes (3, 4, 5). However, there is sparse data on the joint contributions of these risk factors on outcomes. It is well documented that prevention of CVD is best achieved by a comprehensive approach targeted at improving multiple cardiovascular risk factors (6).

Elevated blood pressure or hypertension (defined as systolic and/or diastolic hypertension) is a key intermediate modifiable phenotype for CVD development (7). Based on previous studies including the Framingham Heart Study which showed systolic hypertension to be a more important predictor of cardiovascular outcomes (8, 9), systolic blood pressure (SBP) is more commonly considered in the determination of cardiovascular risk (9). It is well documented that elevated SBP is independently associated with an increased risk of CVD (9).

Sauna bathing, a passive heat therapy, is a Finnish traditional activity that is commonly used for relaxation and pleasure. (10) There is increasing evidence on the health benefits of frequent sauna bathing. Both epidemiological and intervention studies suggest that frequent Finnish sauna bathing may be protective of several adverse health outcomes (11, 12, 13, 14, 15, 16, 17). Higher frequency of sauna bathing is associated with reduced risk of cardiovascular outcomes such as hypertension (18), CVD mortality (19), stroke (20), dementia (21), as well as all-cause mortality (19). Frequent sauna bathing is a strong beneficial lifestyle habit which may potentiate the effects of protective risk factors such as physical fitness (17, 22, 23, 24) or attenuate or offset the adverse effects of other risk factors. We have previously shown that high frequency of sauna bathing (FSB) can offset the increased risk of pneumonia due to inflammation or low socioeconomic status (SES) (25, 26). We hypothesize that there exists a clinically important interactions between SBP levels, sauna bathing frequency, and CVD mortality and, it may be possible that high FSB could mitigate CVD mortality risk among adults with elevated resting SBP.

In this context, using a population-based prospective cohort comprising 2,575 middle-aged to older Finnish men, we aimed to (i) evaluate the independent prospective associations of SBP and FSB with the risk of CVD mortality and (ii) examine the interactions between SBP and FSB with the risk of CVD mortality.

## Methods

Study participants in this analysis were part of the Kuopio Ischemic Heart Disease (KIHD) study, an ongoing population-based prospective cohort study that was designed to investigate risk factors for atherosclerotic CVD. The cohort comprised a representative sample of men aged 42–61 yr recruited from Kuopio or its surrounding rural communities in eastern Finland. The study design, recruitment methods and assessment of risk markers have been described previously (20, 22). Recruitment, screening and baseline assessments were carried out between March 1984 and December 1989. The study protocol was approved by the Research Ethics Committee of the University of Eastern Finland and written informed consent was obtained from all study participants. Resting blood pressure was measured between 8:00 and 10:00 AM with a random-zero sphygmomanometer. After a supine rest of 5-minutes, blood pressure was measured three times in supine position, once in a standing position, and twice in a sitting position with 5-minute intervals, and the arithmetic mean of all available measurements was taken (27). The FSB was assessed based on a traditional Finnish sauna which has air with a relative humidity of 10 to 20%. Sauna bathing habits were assessed by a self-administrated questionnaire which included assessment of the weekly frequency and duration of sauna sessions (20, 22, 23, 28). We included all CVD deaths that occurred from study entry through to 2018 (19, 29). Cox proportional hazards models were used to calculate multivariable-adjusted hazard ratios (HRs) with 95% CIs for CVD mortality. The models were progressively adjusted for age, body mass index (BMI), smoking status, total cholesterol, high-density lipoprotein cholesterol (HDL-C), prevalent type 2 diabetes (T2D) and coronary heart disease (CHD), use of antihypertensive medication, alcohol consumption, physical activity, SES, high sensitivity C-reactive protein (hsCRP) and mutual adjustment for each exposure. The selection of covariates was based on their roles as traditional risk factors for CVD, previously published associations with CVD in the KIHD study (22, 28, 29), or their potential as confounders based on known associations with CVD outcomes and observed associations with the exposures using the available data (30). To maintain consistency with blood pressure guidelines (31), resting SBP was categorized as normal and high (<140 and ≥140 mmHg, respectively). Systolic blood pressure was also modeled as a continuous variable given the linear dose-response relationship between SBP and adverse vascular outcomes (32). Our previous studies of the associations of sauna bathing with adverse outcomes have demonstrated that sauna bathing sessions of 3 or more per week are substantially protective of these outcomes (20, 22, 28, 33, 34), hence, FSB was categorized as low and high (designated as ≤ 2 and 3–7 sauna sessions per week, respectively). To evaluate joint associations, study participants were classified into four groups according to the above defined categories of SBP and FSB: normal SBP-high FSB; normal SBP-low FSB; high SBP-high FSB; and high SBP-low FSB. We evaluated interactions between SBP on both the additive and multiplicative scales in relation to CVD mortality, as described previously (35). Additive interactions were assessed using the “relative excess risk due to interaction” (RERI), computed for binary variables as RERIHR =HR11-HR10-HR01+1 (36), where HR11 is the HR of the outcome (i.e., CVD mortality) if both risk factors (high SBP and low FSB) are present, HR10 is the HR of the outcome if one risk factor is present and the other is absent, with HR01 being vice versa. The RERI has been demonstrated as the best choice among measures of additive interaction using a proportional hazards model (36). However, we also estimated other measures of additive interaction: attributable proportion (AP)=(RERI/HR11) and synergy index (S)=[(HR11 − 1)/(HR10 − 1)+(HR01 − 1)]. The Stata code -ic- which implements the procedure described in Hosmer & Lemeshow (37), was used to generate these measures, their corresponding 95% CIs and two-tailed tests for no interaction. Multiplicative interactions were assessed using the ratio of HRs=HR11/(HR10xHR01) (36). In the absence of interaction, RERI=0, AP=0, S=1 and the ratio of HRs=1. All statistical analyses were conducted using Stata version MP 17 (Stata Corp, College Station, Texas).

## Results

**Figure 1:**
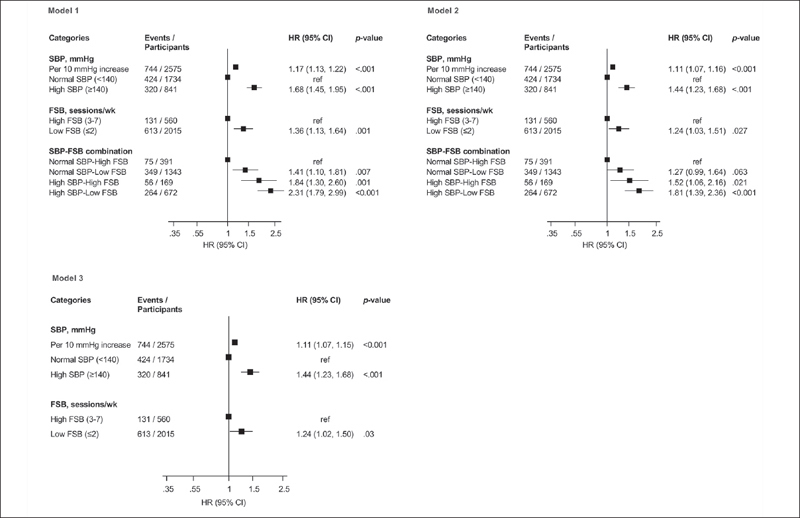
Separate and joint associations of systolic blood pressure and frequency of sauna bathing with the risk of cardiovascular disease mortality CI: confidence interval; FSB: frequency of sauna bathing; HR: hazard ratio; ref: reference; SBP: systolic blood pressure; Model 1: Adjusted for age; Model 2: Model 1 plus body mass index, total cholesterol, high-density lipoprotein cholesterol, smoking status, history of type 2 diabetes, history of coronary heart disease, use of antihypertensive medication, alcohol consumption, physical activity, socioeconomic status, and high sensitivity C-reactive protein; Model 3: Model 2 plus mutual adjustment for each exposure

The overall mean (standard deviation) age and SBP of men at baseline was 53 (5) yr and 134 (17) mmHg, respectively. The median (interquartile range, IQR) FSB was 2 (1–2) sessions/week (Table 1). During a median (IQR) follow-up of 27.8 (18.4–31.1) yr, 744 CVD deaths occurred. Compared with men with normal SBP, those with high SBP had an increased risk of CVD mortality following adjustment for age 1.68 (95% CI: 1.45–1.95) (Figure 1-Model 1), which was minimally attenuated to 1.44 (95% CI: 1.23–1.68) on further adjustment for BMI, smoking status, total cholesterol, HDL-C, prevalent T2D and CHD, use of antihypertensive medication, alcohol consumption, physical activity, SES, and hsCRP (Figure 1-Model 2). When SBP was modeled per 10 mmHg increase, there was evidence of an association. On adjustment for the covariates in Model 2, low FSB was associated with an increased risk of CVD mortality compared with high FSB 1.24 (95% CI: 1.03–1.51) (Figure 1-Model 2). The associations persisted on mutual adjustment for each exposure (Figure 1-Model 3).

**Table 1:** Baseline characteristics of study participants (N=2575)

**Characteristics**	**Overall (N=2575) Mean (SD) or median (IQR)**	**No CVD death (N=1831) Mean (SD) or median (IQR)**	**CVD death (N=744) Mean (SD) or median (IQR)**
Systolic blood pressure, mmHg	134 (17)	132 (16)	138 (18)
Frequency of sauna bathing (sessions/week)	2(1–2)	2(1–2)	2 (1–2)
Questionnaire/Prevalent conditions			
Age, yr	53 (5)	52 (5)	55 (4)
Alcohol consumption, g/wk	31.8 (6.3–91.5)	31.3 (6.5–89.5)	32.0 (4.8–96.0)
Socioeconomic status	8.49 (4.23)	8.11 (4.26)	9.44 (4.01)
Current smoking, n (%)	814 (31.6)	536 (29.3)	278 (37.4)
History of hypertension, n (%)	778 (30.2)	477 (26.1)	301 (40.5)
History of type 2 diabetes, n (%)	104 (4.0)	48 (2.6)	56 (7.5)
History of coronary heart disease, n (%)	649 (25.2)	368 (20.1)	281 (37.8)
Use of antihypertensive medication, n (%)	576 (22.4)	313 (17.1)	263 (35.4)
Physical measurements			
Body mass index, kg/m^2^	26.9 (3.6)	26.6 (3.4)	27.7 (3.9)
Diastolic blood pressure, mmHg	89 (11)	88 (10)	90 (10)
Physical activity (KJ/day)	1204 (628–2000)	1204 (651–1994)	1208 (586–2038)
Blood biomarkers			
Total cholesterol, mmol/l	5.91 (1.08)	5.83 (1.04)	6.10 (1.15)
HDL-C, mmol/l	1.29 (0.30)	1.30 (0.30)	1.26 (0.30)
Fasting plasma glucose, mmol/l	5.36 (1.27)	5.25 (1.04)	5.61 (1.67)
High sensitivity C-reactive protein, mg/l	1.29 (0.71–2.48)	1.16 (0.65–2.22)	1.68 (0.92–3.24)

BMI, body mass index; CHD, coronary heart disease; CVD, cardiovascular disease; HDL-C, high-density lipoprotein cholesterol; IQR, interquartile range; SD, standard deviation

Compared with men with normal SBP-high FSB, multivariable analysis showed that high SBP-low FSB was associated with an increased risk of CVD death 1.81 (95% CI: 1.39–2.36), with attenuated but persisting evidence of an association between high SBP-high FSB and CVD mortality risk 1.52 (95% CI: 1.06–2.16) (Figure 1-Model 2). Interaction analysis showed the RERI=0.02 (95% CI: −0.51, 0.55; p=.95), AP=0.01 (95% CI: −0.28, 0.30; p=.95), S=1.02 (95% CI: 0.52, 2.00; p=.95), and the ratio of HRs=0.94 (95% CI: 0.57, 1.30; p=.16), suggesting the presence of positive additive and negative multiplicative interactions but the estimates were not significant. Given that the American College of Cardiology/American Heart Association guidelines defines hypertension using a SBP cutoff of ≥130 mmHg (38), we conducted a subsidiary analysis in which SBP was categorized as low and high (<130 and ≥130 mmHg, respectively) and we re-evaluated the joint associations of SBP and FSB with CVD mortality risk. Whereas there was evidence of an association in men with high SBP-low FSB 1.58 (95% CI: 1.16–2.17), it was attenuated to null in men with high SBP-high FSB 1.11 (95% CI: 0.77–1.61) (Table 2).

**Table 2:** Separate and joint associations of systolic blood pressure and frequency of sauna bathing with risk of CVD mortality using a systolic blood pressure cutoff of 130 mmHg

**Exposure**	**Events/Total**	**Model 1**		**Model 2**		**Model 3**	
		**HR (95% CI)**	**p-value**	**HR (95% CI)**	**p-value**	**HR (95% CI)**	**p-value**
SBP, mmHg							
Normal SBP (<130)	242/1117	ref		ref		ref	
High SBP (≥130)	502/1458	1.67 (1.43–1.95)	<.001	1.49 (1.27–1.75)	<.001	1.51 (1.28–1.77)	<.001
FSB, sessions/week							
High FSB (3–7)	311/560	ref		ref		ref	
Low FSB (≤2)	613/2015	1.36 (1.13–1.64)	.001	1.24 (1.03–1.51)	.027	1.24 (1.02–1.50)	.030
SBP-FSB combination							
Normal SBP-High FSB	46/242	ref		ref	ref	NA	
Normal SBP-Low FSB	196/875	1.16 (0.84–1.61)	.35	0.99 (0.71–1.38)	.95	NA	
High SBP- High FSB	85/318	1.36 (0.95–1.95)	.090	1.11 (0.77–1.61)	.57	NA	
High SBP-Low FSB	417/1140	2.05 (1.51–2.78)	<.001	1.58 (1.16–2.17)	.004	NA	

CI: confidence interval; CVD: cardiovascular disease; FSB: frequency of sauna bathing; HR: hazard ratio; ref: reference; NA,:not applicable; SBP: systolic blood pressure; Model 1: Adjusted for age; Model 2: Model 1 plus body mass index, total cholesterol, high-density lipoprotein cholesterol, smoking status, history of type 2 diabetes, history of coronary heart disease, use of antihypertensive medication, alcohol consumption, physical activity, socioeconomic status, and high sensitivity C-reactive protein; Model 3: Model 2 plus mutual adjustment for each exposure

## Discussion

In line with previous reports (9, 28), high SBP (defined as ≥130 or ≥140 mmHg) and low FSB were each independently associated with increased CVD mortality in this cohort of middle-aged and older Finnish men. The associations persisted on mutual adjustment for each exposure. Our current findings based on the prospective associations of the SBP-FSB phenotype (using a SBP cutoff of 140 mmHg) with the risk of CVD mortality showed that the risk was increased in men with elevated SBP and low FSB, with an attenuated but persisting risk in men with elevated SBP who engaged in frequent sauna baths. When a SBP cutoff of 130 mmHg was used, the risk of CVD mortality was offset in men with high SBP-high FSB. The overall findings suggest that frequent sauna baths may offset the increased risk of CVD mortality in men with high-normal SBP (defined as ≥130 mmHg) but not elevated SBP (defined as ≥140 mmHg). In interaction analysis, there was modest evidence that the association between the combined exposures (i.e., high SBP and low FSB) and CVD mortality risk exceeded the sum of their associations considered separately.

Though high blood pressure or hypertension is a major risk factor for CVD globally (7), systolic hypertension is considered a more important determinant of cardiovascular outcomes than diastolic hypertension (8). The relationship between SBP and CVD has been described as strong, graded and causal (6, 39). Sauna bathing (a passive heat therapy) has been reported to produce physiological responses and adaptations that are similar to those produced by moderate or high intensity physical activity (40). Pathways proposed to underlie the associations between passive heat exposure and decreased risk of CVD include beneficial modulation of cardiovascular risk factors such as blood pressure, lipids, and natriuretic peptides; reduction in oxidative stress and low-grade systemic inflammation; improvement in endothelial function; beneficial modulation of the cardiac autonomic nervous system; improved arterial stiffness, arterial compliance, and intima media thickness; and overall improvement in cardiovascular function (10). In our previous prospective study, we showed that life-long sauna bathing was associated with a reduced risk of incident hypertension (18). Based on the current findings and previous evidence of the ability of frequent sauna baths to mitigate the adverse effects of other risk factors (25, 26), one may conclude that the protective effects exerted by frequent sauna baths are large enough to offset the adverse effects of high-normal SBP, but the effects of sauna are modest in the presence of elevated SBP. Further investigations are required in the form of intervention and mechanistic studies.

These findings add to the emerging evidence on the ability of frequent sauna exposure to prevent some adverse health outcomes and also attenuate or mitigate the adverse effects of other risk factors (25, 26). Regular physical activity plays a pivotable role in the management of high blood pressure (38), the major risk factor for CVD; regular aerobic exercise results in mean reductions in blood pressure of 5–7 mmHg among individuals with hypertension and these reductions translate to a reduced risk of CVD of 20–30% (41). Given that recent evidence suggests that regular heat therapy is able to lower blood pressure to a degree comparable to that of physical activity (42), this suggests that adding frequent sauna bathing to regular physical activity may yield substantial benefits on blood pressure and cardiovascular risk. Indeed, in a recent randomized controlled trial, we showed that sauna bathing had a substantial supplementary effect on levels of cardiorespiratory fitness, SBP and total cholesterol when combined with exercise (17); eight weeks of regular sauna bathing sessions combined with exercise produced a mean reduction in SBP of 8 mmHg as compared to exercise training alone in individuals with at least one traditional cardiovascular risk factor (17).

Some may argue that given that sauna bathing is more commonly used in Nordic countries, the potential beneficial implications may not be applicable in other populations. However, several definitive epidemiological and interventional investigations have reported robust evidence on the health benefits of sauna bathing over the last decade; furthermore, sauna bathing is now becoming a common lifestyle activity on a global scale (43, 44). Sauna use has a good safety profile, and most people in generally good health can tolerate it without significant risks (10). Individuals at risk of orthostatic hypotension should exercise caution during sauna sessions because of the pronounced blood pressure lowering effect, which may also occur during the recovery period after a sauna session. Contraindications to sauna use have included unstable angina pectoris, recent myocardial infarction, uncontrolled hypertension, decompensated heart failure or severe aortic stenosis (10). Consistent with physical activity and exercise recommendations, there is enough evidence to justify the promotion and wider use of sauna among the population.

The current study is novel, being the first evaluation of the clinically significant interaction between SBP, sauna bathing and CVD mortality. Other strengths include formal investigation of the interactions between SBP and FSB in relation to CVD mortality, the use of a population-based prospective cohort design comprising a relatively large sample homogeneous sample of men, the long-term follow-up duration of the cohort, and availability of a comprehensive panel of potential confounders for adjustment. The limitations are mostly inherent to the study design and included the lack of generalisability of the results to women, misclassification bias due to self-reported sauna habits, lack of data on possible changes in the use of medication during the long follow-up and potential biases of observational cohort designs such as residual confounding, reverse causation, and regression dilution bias.

## Conclusions

Both SBP and FSB are each associated with the risk of CVD mortality, independently of several established risk factors. There exists an interplay between SBP, sauna bathing, and CVD mortality risk — there may be some interactive effects of blood pressure and sauna bathing in relation to the risk of cardiovascular mortality and frequent sauna baths may offset the increased risk of CVD mortality in men with high-normal SBP but not elevated SBP.

## Funding

Open access funding provided by University of Eastern Finland (UEF) including Kuopio University Hospital.

## Conflicts of interest:

None.
